# Immunophenotyping of COVID-19 and influenza highlights the role of type I interferons in development of severe COVID-19

**DOI:** 10.1126/sciimmunol.abd1554

**Published:** 2020-07-10

**Authors:** Jeong Seok Lee, Seongwan Park, Hye Won Jeong, Jin Young Ahn, Seong Jin Choi, Hoyoung Lee, Baekgyu Choi, Su Kyung Nam, Moa Sa, Ji-Soo Kwon, Su Jin Jeong, Heung Kyu Lee, Sung Ho Park, Su-Hyung Park, Jun Yong Choi, Sung-Han Kim, Inkyung Jung, Eui-Cheol Shin

**Affiliations:** 1 Graduate School of Medical Science and Engineering, Korea Advanced Institute of Science and Technology (KAIST), Daejeon 34141, Republic of Korea.; 2 Department of Biological Sciences, KAIST, Daejeon 34141, Republic of Korea.; 3 Department of Internal Medicine, Chungbuk National University College of Medicine, Cheongju 28644, Republic of Korea.; 4 Department of Internal Medicine, Severance Hospital, Yonsei University College of Medicine, Seoul 03722, Republic of Korea.; 5 The Center for Epidemic Preparedness, KAIST Institute, Daejeon 34141, Republic of Korea.; 6 Department of Infectious Diseases, Asan Medical Center, University of Ulsan College of Medicine, Seoul 05505, Republic of Korea.; 7 School of Life Sciences, Ulsan National Institute of Science & Technology (UNIST), Ulsan 44919, Republic of Korea.

## Abstract

Although most SARS-CoV-2-infected individuals experience mild coronavirus disease 2019 (COVID-19), some patients suffer from severe COVID-19, which is accompanied by acute respiratory distress syndrome and systemic inflammation. To identify factors driving severe progression of COVID-19, we performed single-cell RNA-seq using peripheral blood mononuclear cells (PBMCs) obtained from healthy donors, patients with mild or severe COVID-19, and patients with severe influenza. Patients with COVID-19 exhibited hyper-inflammatory signatures across all types of cells among PBMCs, particularly up-regulation of the TNF/IL-1β-driven inflammatory response as compared to severe influenza. In classical monocytes from patients with severe COVID-19, type I IFN response co-existed with the TNF/IL-1β-driven inflammation, and this was not seen in patients with milder COVID-19. Interestingly, we documented type I IFN-driven inflammatory features in patients with severe influenza as well. Based on this, we propose that the type I IFN response plays a pivotal role in exacerbating inflammation in severe COVID-19.

## Teaser

Single-cell RNA sequencing of blood immune cells reveals type I interferon-associated hyper-inflammation in severe COVID-19.

## INTRODUCTION

Currently, severe acute respiratory syndrome coronavirus 2 (SARS-CoV-2), which causes coronavirus disease 2019 (COVID-19), is spreading globally (*1*, *2*), and the World Health Organization (WHO) has declared it a pandemic. As of June 2, 2020, more than 6.1 million confirmed cases and more than 376,000 deaths have been reported worldwide (*3*).

SARS-CoV-2 infection usually results in a mild disease course with spontaneous resolution in the majority of infected individuals (*4*). However, some patients, particularly elderly patients develop severe COVID-19 infection that requires intensive care with mechanical ventilation (*4*, *5*). The mortality rate for COVID-19 in Wuhan, China, is estimated to be 1.4% (*5*). Although this rate is lower than that of severe acute respiratory syndrome (SARS) and Middle East respiratory syndrome (MERS), which are caused by other human pathogenic coronaviruses (*6*), it is much higher than that of influenza, a common respiratory viral disease requiring hospitalization and intensive care in severe cases.

In severe cases of COVID-19, a hyper-inflammatory response, also called a cytokine storm, has been observed and is suspected of causing the detrimental progression of COVID-19 (*7*). Circulating levels of pro-inflammatory cytokines, including TNF and IL-6, are increased in severe cases (*8*). Gene expression analyses have also shown that IL-1-related pro-inflammatory pathways are highly up-regulated in severe cases (*9*). In a murine model of SARS-CoV infection, a delayed, but considerable type I IFN (IFN-I) response promotes the accumulation of monocytes-macrophages and the production of pro-inflammatory cytokines, resulting in lethal pneumonia with vascular leakage and impaired virus-specific T-cell responses (*10*).

Immune dysfunction is also observed in patients with COVID-19. In severe cases, the absolute number of T cells is reduced (*8*, *11*), and the T cells exhibit functional exhaustion with the expression of inhibitory receptors (*12*, *13*). However, hyper-activation of T cells as reflected in the up-regulation of CD38, HLA-DR, and cytotoxic molecules was also reported in a lethal case of COVID-19 (*14*). Immune dysfunction in patients with severe COVID-19 has been attributed to pro-inflammatory cytokines (*15*).

In the present study, we performed single-cell RNA-seq (scRNA-seq) using peripheral blood mononuclear cells (PBMCs) to identify factors associated with the development of severe COVID-19 infection. By comparing COVID-19 and severe influenza, we report that the TNF/IL-1β-driven inflammatory response was dominant in COVID-19 across all types of cells among PBMCs, whereas the up-regulation of various interferon-stimulated genes (ISGs) was prominent in severe influenza. When we compared the immune responses from patients with mild and severe COVID-19 infections, we found that classical monocytes from severe COVID-19 exhibit IFN-I-driven signatures in addition to TNF/IL-1β-driven inflammation.

## RESULTS

### Single-cell transcriptomes of PBMCs from patients with COVID-19 and influenza

PBMCs were collected from healthy donors (n=4), hospitalized patients with severe influenza (n=5), and patients with COVID-19 of varying clinical severity, including severe, mild, and asymptomatic (n=8). PBMCs were obtained twice from three (the subject C3, C6, and C7) of the eight COVID-19 patients at different time points during hospitalization. PBMC specimens from COVID-19 patients were assigned to severe or mild COVID-19 groups according to the National Early Warning Score (NEWS; mild < 5, severe ≥ 5) evaluated on the day of whole blood sampling (*16*). In NEWS scoring, respiratory rate, oxygen saturation, oxygen supplement, body temperature, systolic blood pressure, heart rate, and consciousness were evaluated (*16*). Severe influenza was defined when hospitalization was required irrespective of NEWS score. Patients with severe influenza were enrolled from December 2015 to April 2016, prior to the emergence of COVID-19. The severe COVID-19 group was characterized by significantly lower lymphocyte count and higher serum level of C-reactive protein than the mild COVID-19 group on the day of blood sampling (Fig. S1A). Multiplex real-time PCR for N, RdRP, and E genes of SARS-CoV-2 was performed, and there was no statistical difference in Ct values for all three genes between two groups (Fig. S1B). Demographic information is provided with experimental batch of scRNA-seq in Table S1 and clinical data in Table S2 and S3.

Employing the 10X Genomics scRNA-seq platform, we analyzed a total of 59,572 cells in all patients after filtering the data with stringent high quality, yielding a mean of 6,900 UMIs per cell and detecting 1,900 genes per cell on average (Table S4). The transcriptome profiles of biological replicates (PBMC specimens in the same group) were highly reproducible (Fig. S1C), ensuring the high quality of the scRNA-seq data generated in this study.

**Fig. 1:**
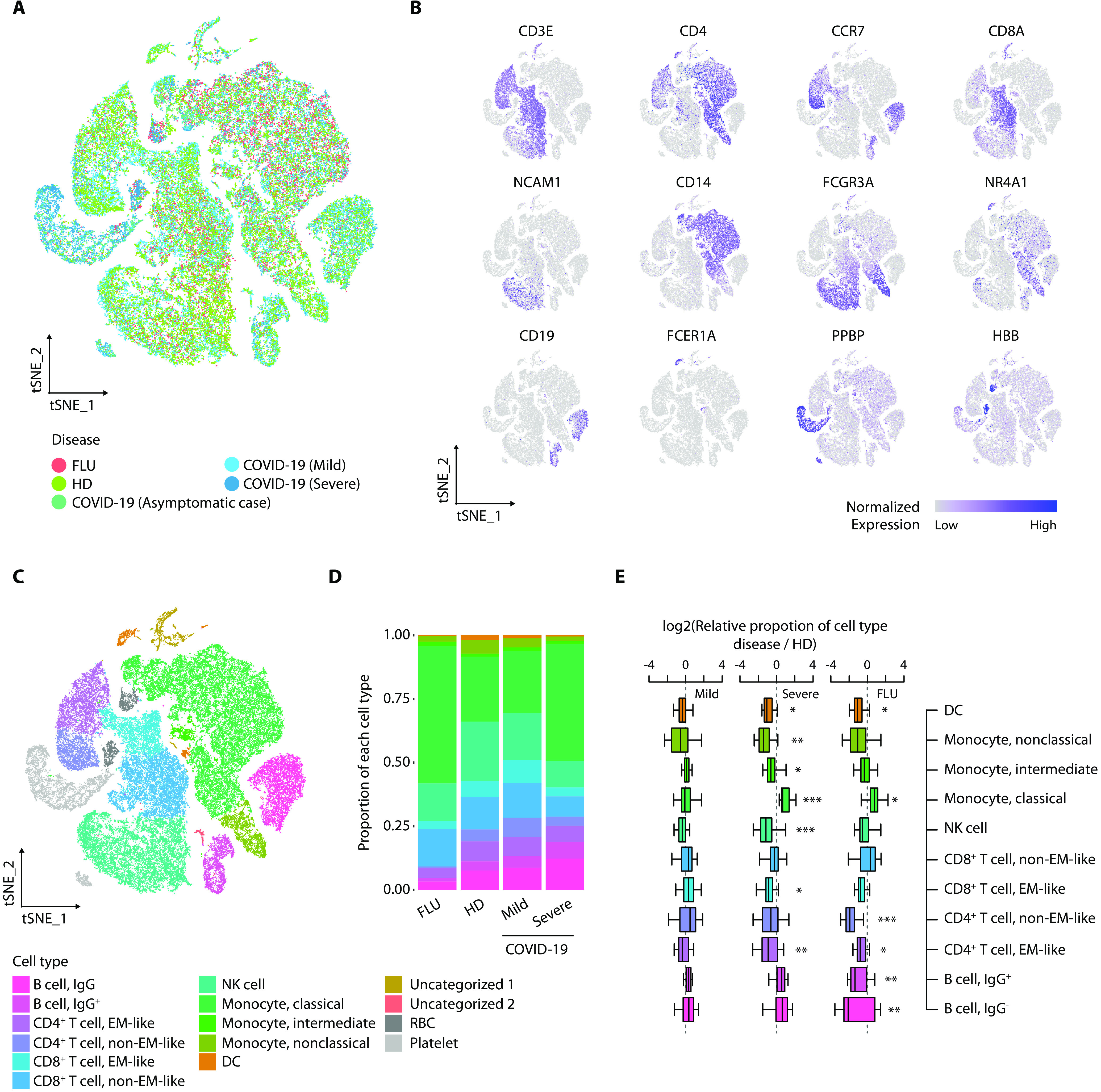
Single cell transcriptomes of PBMCs from COVID-19 and influenza patients. (A) tSNE projections of 59,572 PBMCs from healthy donors (HDs) (4 samples, 17,590 cells), severe influenza (FLU) patients (5 samples, 10,519 cells), COVID-19 patients (asymptomatic: 1 sample, 4,425 cells; mild COVID-19: 4 samples, 16,742 cells; severe COVID-19: 6 samples, 10,296 cells) colored by group information. (B) Normalized expression of known marker genes on a tSNE plot. (C) tSNE plot colored by annotated cell types. EM: effector memory, NK cell: natural killer cell, DC: dendritic cell, RBC: red blood cell. (D) Proportion of cell types in each group excluding ‘Uncategorized 1’, ‘Uncategorized 2’, ‘RBC’, and ‘Platelet’. The colors indicate cell type information. (E) Boxplots showing the fold enrichment in cell type proportions from mild COVID-19 (n=4), severe COVID-19 (n=6), and FLU (n=5) patients compared to the HD group (mild COVID-19 vs. HD: n=16, severe COVID-19 vs. HD: n=24, FLU vs. HD: n=20). For the boxplots, the box represents the interquartile range (IQR) and the whiskers correspond to the highest and lowest points within 1.5 × IQR. ‘Uncategorized 1’ (relatively high UMIs per cells and presence of multiple marker genes), ‘Uncategorized 2’ (B cell-like and high expression of ribosomal protein genes), ‘RBC’, and ‘Platelet’ were excluded. Two-sided Kolmogorov–Smirnov (KS) tests were conducted for each cell type between the disease and HD group. *p<0.05, **p<0.01, and ***p<0.001.

To examine the host immune responses in a cell type-specific manner, we subjected 59,572 cells to t-distributed stochastic neighbor embedding (tSNE) based on highly variable genes using the Seurat package (*17*) and identified 22 different clusters unbiased by patients or experimental batches of scRNA-seq (Fig. 1A, Fig. S1D). These clusters were assigned to 13 different cell types based on well-known marker genes and two uncategorized clusters (Fig. 1B and C, and Table S5). In downstream analysis, we only focused on 11 different immune cell types, including IgG^-^ B cell, IgG^+^ B cell, effector memory (EM)-like CD4^+^ T cell, non-EM-like CD4^+^ T cell, EM-like CD8^+^ T cell, non-EM-like CD8^+^ T cell, natural killer (NK) cell, classical monocyte, intermediate monocyte, non-classical monocyte, and dendritic cell (DC) after excluding platelets, red blood cells (RBCs), and two uncategorized clusters. The subject C8 (asymptomatic case) was also excluded due to a lack of replicates. In hierarchical clustering, most transcriptome profiles from the same cell type tended to cluster together, followed by disease groups, suggesting that both immune cell type and disease biology, rather than technical artifacts, are the main drivers of the variable immune transcriptome (Fig. S1E).

As a feature of immunological changes, we investigated the relative proportions of immune cells among PBMCs in the disease groups compared to the healthy donor group (Fig. 1D and E, and Fig. S1F). Unlike the limited changes in mild COVID-19, significant changes were observed in both influenza and severe COVID-19 across multiple cell types among PBMCs. In severe COVID-19, the proportion of classical monocytes significantly increased whereas those of DCs, non-classical monocytes, intermediate monocytes, NK cells, EM-like CD8^+^ T cells, and EM-like CD4^+^ T cells significantly decreased (Fig. 1E). In severe influenza, the proportion of classical monocytes significantly increased whereas those of DCs, non-EM-like CD4^+^ T cells, EM-like CD4^+^ T cells, IgG^+^ B cells, and IgG^-^ B cells significantly decreased. We validated the proportions of immune cell subsets from scRNA-seq by flow cytometry analysis. The relative proportions of total lymphocytes, B cells, CD4^+^ T cells, CD8^+^ T cells, NK cells, and total monocytes from scRNA-seq significantly correlated with those from flow cytometry analysis (Fig. S1G).

### Transcriptional signatures associated with COVID-19

**Fig. 2:**
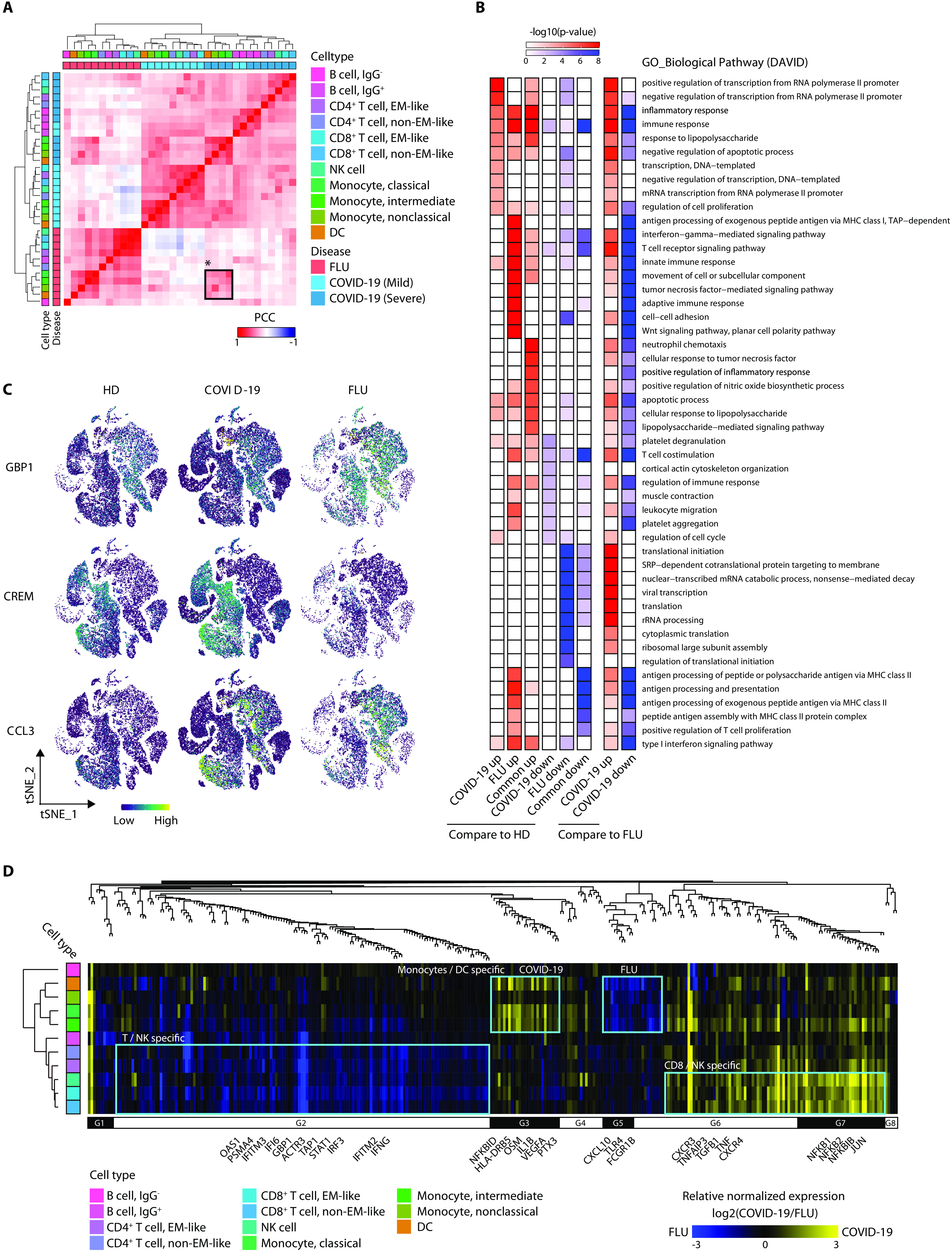
Immune landscape of COVID-19. (A) Hierarchical clustering using the Pearson correlation coefficient (PCC) of a normalized transcriptome between diseases in cell type resolution (n = 33). The color intensity of the heat map indicates the PCC values. The color bars above the heat map indicate the cell type and disease group. The black box indicates the cell types that highly correlate between the severe COVID-19 and FLU groups. (B) Illustration of the enrichment p-values for the select GO biological pathways (n = 49) of differentially expressed genes (DEGs) in COVID-19 and FLU patients (left 6 columns: DEGs for COVID-19 and FLU groups compared to HD, right 2 columns: DEGs between COVID-19 and FLU groups). (C) tSNE plot of representative gene expression patterns for *GBP1* (FLU specific), *CREM* (COVID-19 specific), and *CCL3* (COVID-19/FLU common). (D) Top, dendrogram from WGCNA analysis performed using relative normalized gene expression between the COVID-19 and FLU groups for the genes belonging to the select biological pathways in (B) (n=316). Bottom, heat map of relative normalized gene expression between the COVID-19 and FLU groups. The color bar (left) indicates cell type information clustered by hierarchical clustering based on the PCC for relative normalized gene expression. Modularized gene expression patterns by WGCNA are shown together (G1, n=10; G2, n=147; G3, n=27; G4, n=17; G5, n=12; G6, n=64; G7, n=34; G8, n=5).

In order to compare the effect of infection between diseases, we performed hierarchical clustering based on relative gene expression changes against the healthy donor group. Unexpectedly, all types of cells among PBMCs were clustered together according to the disease groups instead of cell-types (Fig. 2A). Further investigation of the variable genes based on K-means clustering supported COVID-19-specific up- or down-regulated gene expression patterns across all types of cells among PBMCs (Fig. S2A). These results indicate that, in COVID-19, peripheral blood immune cells may be influenced by common inflammatory mediators regardless of cell type. Despite distinct transcriptional signatures between COVID-19 and influenza, severe COVID-19 and influenza shared transcriptional signatures in all types of monocytes and DCs (black boxed region in Fig. 2A), possibly reflecting common mechanisms underlying the innate immune responses in severe influenza and severe COVID-19.

Next, we sought to identify relevant biological functions in disease-specific up- or down-regulated genes in terms of the GO biological pathways. First, we combined both mild and severe COVID-19 as a COVID-19 group and identified disease-specific changes in genes for each cell type compared to the healthy donor group using model-based analysis of single cell transcriptomics (MAST) (*18*). *NFKB1*, *NFKB2*, *IRF1*, and *CXCR3* were specifically up-regulated in COVID-19, and *CXCL10*, *STAT1*, *TLR4,* and genes for class II HLA and immunoproteasome subunits were specifically up-regulated in influenza (Table S6). *TNF*, *TGFB1*, *IL1B*, and *IFNG* were commonly up-regulated. When we directly compared COVID-19 and influenza, *NFKB1*, *NFKB2*, and *TNF* were up-regulated in COVID-19, whereas *STAT1*, *TLR4*, and genes for immunoproteasome subunits were up-regulated in influenza. For each group of differentially expressed genes (DEGs), we identified the top 10 enriched GO biological pathways and collected them to demonstrate p-value enrichment in each group of DEGs (Fig. 2B). Both distinct and common biological functions were identified as illustrated by inflammatory response genes being highly active in both COVID-19 and influenza, but genes for transcription factors, including inflammatory factors (i.e., *NFKB1/2,* and *STAT4*) were up-regulated in COVID-19. In contrast, a limited response in genes associated with the IFN-I and -II signaling pathways, T-cell receptor pathways, and adaptive immune response was observed in COVID-19 compared to influenza. Such disease-specific gene expression patterns were exemplified at single cell resolution by *GBP1* (IFN-γ-mediated signaling pathway) being specifically up-regulated in influenza, *CREM* (positive regulation of transcription) being specifically up-regulated in COVID-19, and *CCL3* (inflammatory response) being commonly up-regulated (Fig. 2C and Table S7).

We expanded our analysis in a cell type specific manner by conducting weighted gene correlation network analysis (WGCNA) (*19*) for the collected genes associated with Fig. 2B. We identified several modular expression patterns (Fig. 2D and Table S8). In the COVID-19 group, *NFKB1/2, JUN,* and *TNF* were modularized in CD8^+^ T and NK cells (G6 and G7 in Fig. 2D), and *IL1B, NFKBID,* and *OSM* were modularized in all types of monocytes and DCs (G3 in Fig. 2D). In the influenza group, *GBP1, TAP1, STAT1, IFITM3, OAS1, IRF3,* and *IFNG* were modularized in all types of T cells and NK cells (G2 in Fig. 2D), and *CXCL10* and *TLR4* were modularized in all types of monocytes and DCs (G5 and part of G6 in Fig. 2D). Consistently, the DEGs between COVID-19 and influenza were dominant in CD8^+^ T cells and all types of monocytes (Fig. S2B).

### Distinct subpopulations of CD8^+^ T cells in COVID-19 and influenza

**Fig. 3:**
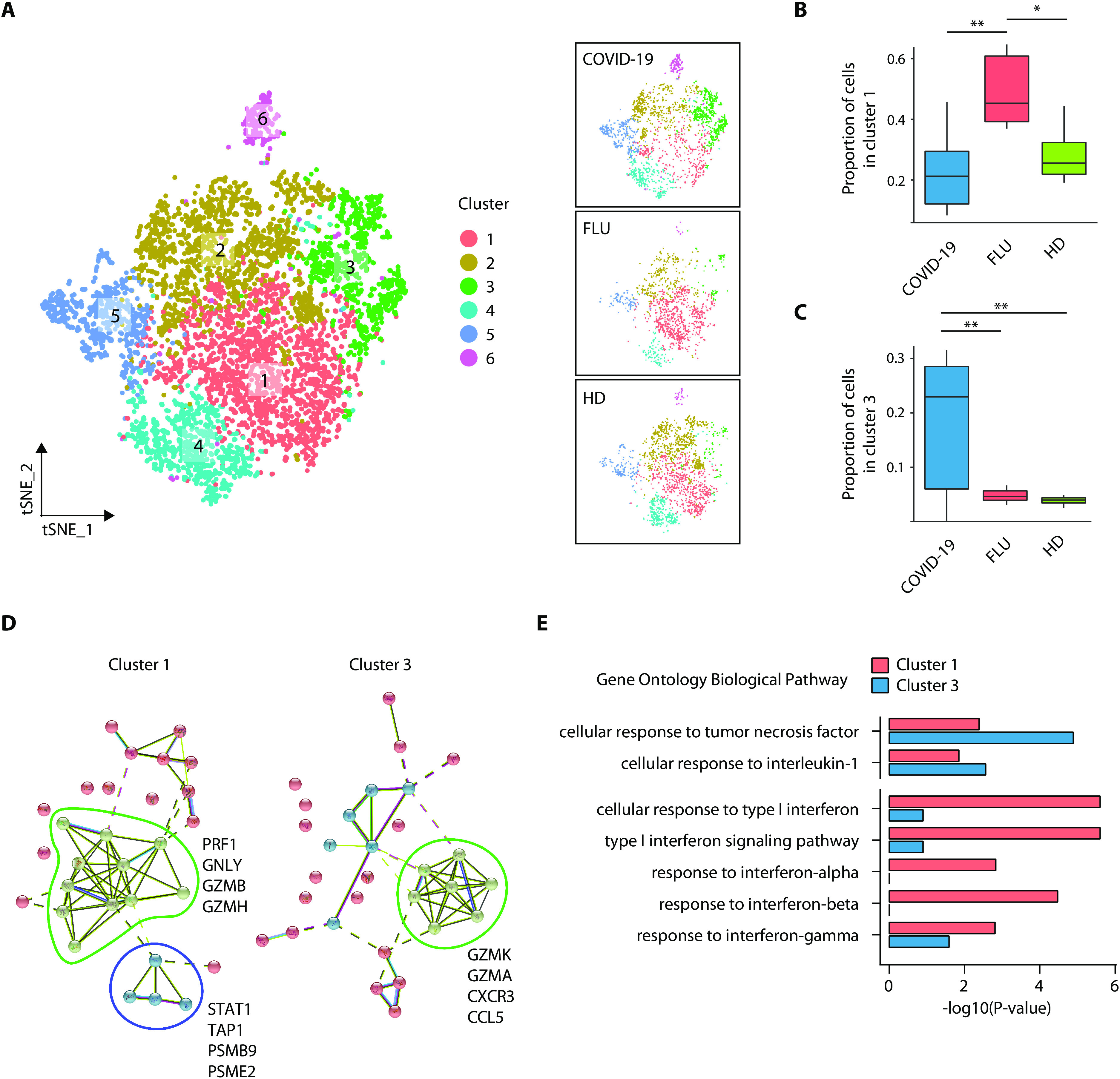
Subpopulation analysis of CD8^+^ T cells. (A) tSNE plot of the non-EM-like CD8^+^ T cell subpopulations in all groups (left, n=6,253), COVID-19 (top right, n=2,653), FLU (middle right, n=1,452), and HD (bottom right, n=2,148) colored by cluster information. (B, C) Boxplots showing the proportion of individual sub-clusters from the non-EM-like CD8^+^ T cell cluster within each group (COVID-19, n=10; FLU, n=5; HD, n=4). The proportions follow normal distribution as tested by the Shapiro-Wilk normality test except the proportion of cluster 3 in the COVID-19 group (p=0.04). Cluster 1 and cluster 3 were highly enriched in the FLU and COVID-19 group, respectively. Two-sided Welch’s *t* test p-values were 4.4E-03 between COVID-19 and FLU in cluster 1, 3.5E-02 between FLU and HD donor in cluster 1, 8.6E-03 between COVID-19 and FLU in cluster 3, and 5.8E-3 between COVID-19 and HD in cluster 3. *p<0.05, **p<0.01. (D) STRING analysis using the top 30 up-regulated genes in cluster 1 (left) and cluster 3 (right). (E) Bar plots showing enrichment p-values of eight representative GO biological pathways for pro-inflammation and interferon in cluster 1 or cluster 3-specific up-regulated genes (cluster 1, n=66; cluster 3, n=183).

To uncover disease-specific transcriptional signatures in CD8^+^ T cells, we performed sub-clustering analysis from EM-like and non-EM-like CD8^+^ T cell clusters using Seurat (*17*). Each disease group-specifically enriched sub-clusters compared to the two other groups were identified in the non-EM-like CD8^+^ T cell cluster (Fig. 3A). Of the six sub-clusters from the non-EM-like CD8^+^ T cell cluster, cluster 1 and cluster 3 were significantly enriched in the influenza and COVID-19 groups, respectively (Fig. 3B and C, and S3A). Clusters with the high expression of *PPBP*, a marker of platelets, were excluded in following analysis (e.g., cluster 6 in Fig. S3A). Intriguingly, up-regulated genes in cluster 1 and cluster 3 were associated with previously defined gene sets for ‘influenza A virus infection’ and ‘SARS-CoV infection’, respectively (Fig. S3B) (*20*). We also found that the cluster 3-specific up-regulated genes reflect activation of immune response, including *CD27, RGS1, CCL5, SELL,* and *RGS10* (Fig. S3C and Table S9). Protein interaction network analysis of selected top 30 up-regulated genes in each cluster based on STRING v11 (*21*) revealed the up-regulation of *PRF1*, *GNLY*, *GZMB*, and *GZMH* in cluster 1 and the up-regulation of *GZMK*, *GZMA*, *CXCR3*, and *CCL5* in cluster 3 (Fig. 3D, green). *STAT1*, *TAP1*, *PSMB9*, and *PSME2*, which are up-regulated preferentially by IFN-γ, were overexpressed only in influenza-specific cluster 1 (Fig. 3D, blue). We validated these data by intracellular staining for granzyme B and PMA/ionomycin-stimulated intracellular cytokine staining for IFN-γ. The percentages of granzyme B^+^ and IFN-γ^+^ cells among CD8^+^ T cells were significantly higher in the influenza group than in the COVID-19 group (Fig. S3D). Of the seven representative GO biological pathways for the pro-inflammatory and IFN responses, pathways for responses to IFN-I and -II were more associated with influenza-specific cluster 1, whereas pathways for the response to TNF or IL-1β were more prominent in COVID-19-specific cluster 3 (Fig. 3E).

### Transcriptional signatures of classical monocytes in COVID-19

**Fig. 4:**
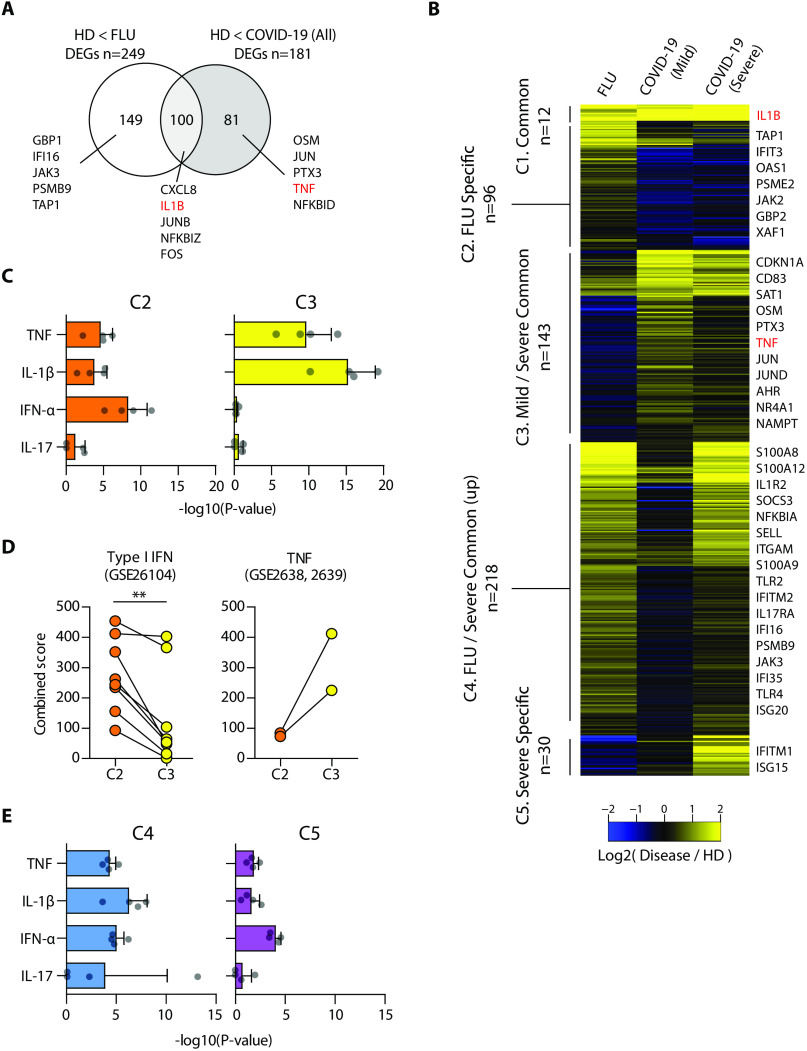
Transcriptome of classical monocytes in COVID-19 patients. (A) Venn diagram of differentially expressed genes (DEGs) in COVID-19 and FLU compared to HD. The representative genes are shown together. (B) K-means clustering of DEGs between all pairs of FLU, mild COVID-19, and severe COVID-19 (n=499). The color indicates the relative gene expression between the diseases and HD. The representative genes are shown together. (C) Bar plots showing the average –log10(p*-*value) values in enrichment analysis using the perturbed genes of four different cell lines listed in L1000 LINCS for up-regulated genes in cluster 2 (C2, left) and cluster 3 (C3, right). Error bars indicate standard deviation. (D) Combined enrichment scores were compared between C2 and C3 for the gene sets of the type I IFN response (left; GSE26104) and TNF response (right; GSE2638, GSE2639). **p<0.01. Each dot indicates an individual subject. (E) Bar plots showing the average –log10(p*-*value) values in the enrichment analysis using the perturbed genes listed of four different cell lines in L1000 LINCS for up-regulated genes in cluster 4 (C4, left) and cluster 5 (C5, right). Error bars indicate standard deviation (C and E).

We performed sub-clustering analysis from all three types of monocyte clusters to find COVID-19-specific sub-clusters. However, there was no COVID-19-specifically enriched sub-cluster (Fig. S4A and B). Next, we further focused on classical monocytes considering their crucial roles for inflammatory responses. We investigated DEGs between influenza and COVID-19 to seek COVID-19-specific transcriptional signatures in classical monocytes (Fig. 4A). Interestingly, *TNF* and *IL1B*, major genes in the inflammatory response, were identified as COVID-19-specific and commonly up-regulated genes, respectively. To better characterize the transcriptional signatures in classical monocytes, we performed K-means clustering of up-regulated genes in at least one disease group compared to the healthy donor group. We identified five different clusters of up-regulation (Fig. 4B and Table S10): genes in cluster 1 are commonly up-regulated in all disease groups, cluster 2 is influenza-specific, cluster 3 is associated with mild/severe COVID-19, cluster 4 is associated with influenza and severe COVID-19, and cluster 5 is severe COVID-19-specific.

We examined each cluster-specific genes by gene set enrichment analysis (GSEA) using cytokine-responsive gene sets originated from each cytokine-treated cells (LINCS L1000 ligand perturbation analysis in Enrichr) (*22*). COVID-19-specific cluster 3 genes were enriched by TNF/IL-1β-responsive genes whereas influenza-specific cluster 2 genes were enriched by IFN-I-responsive genes in addition to TNF/IL-1β-responsive genes (Fig. 4C), indicating that the IFN-I response is dominant in influenza compared to COVID-19. We confirmed this result by analyzing cluster-specific genes with cytokine-responsive gene sets originated from other sources (Fig. 4D). Unexpectedly, cluster 4 and 5 exhibited strong associations with IFN-I-responsive genes, in addition to TNF/IL-1β-responsive genes (Fig. 4E), indicating that severe COVID-19 acquires IFN-I-responsive features in addition to TNF/IL-1β-inflammatory features.

### IFN-I response in addition to TNF/IL-1β inflammatory response in severe COVID-19

**Fig. 5:**
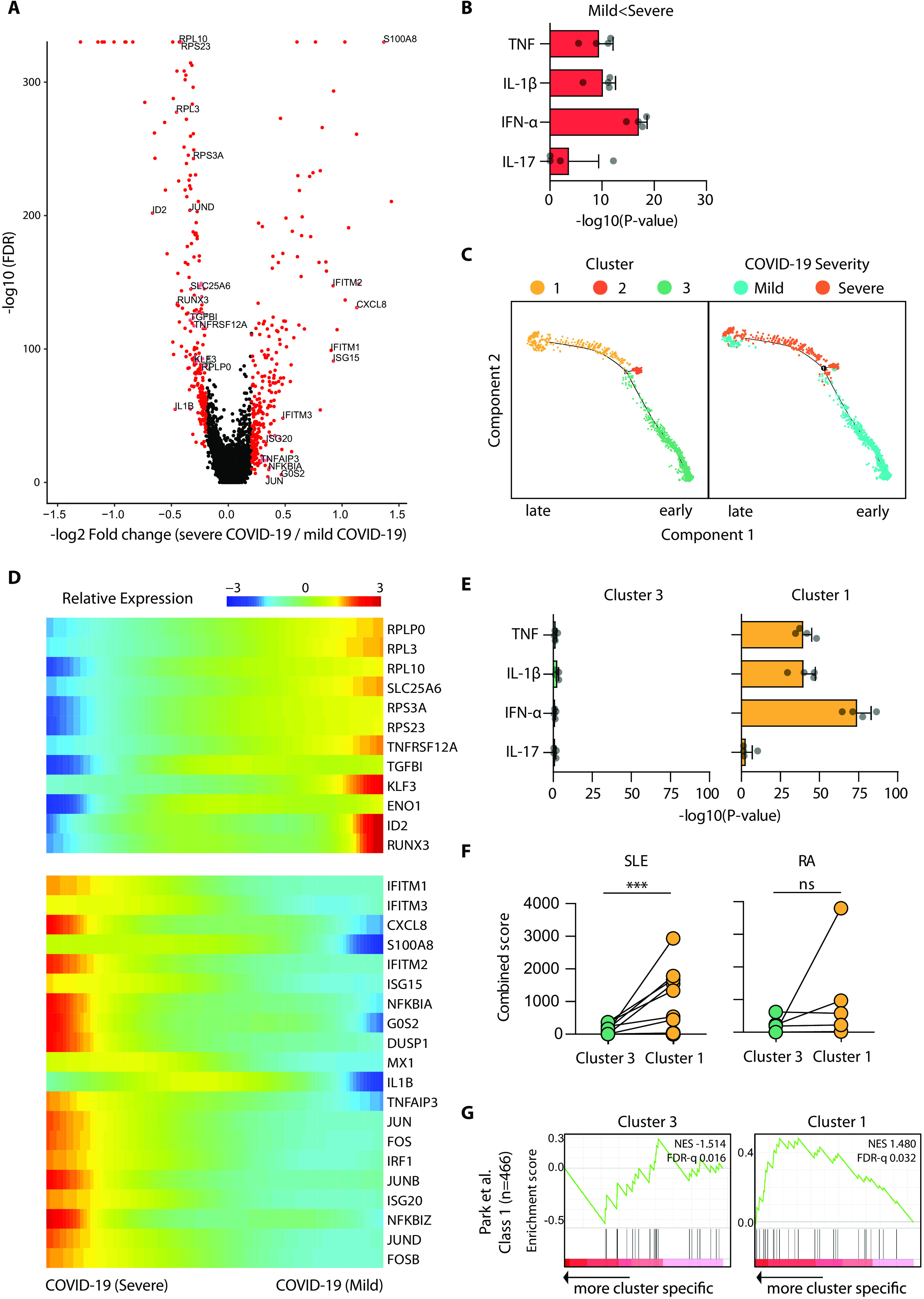
Trajectory analysis of classical monocytes. (A) Volcano plot showing DEGs between mild and severe COVID-19 groups. Each dot indicates individual gene, colored by red when a gene is significant DEG. (B) Bar plot showing the average –log10(p*-*value) values in enrichment analysis using the perturbed genes of four different cell lines listed in L1000 LINCS for up-regulated genes in the severe COVID-19 group. Error bars indicate standard deviation. (C) Trajectory analysis of classical monocytes from specimens obtained at two different time points in a single COVID-19 patient (mild: C7-2, 1,197 cells; severe: C7-1, 631 cells). The color indicates cluster information (left) or the severity of COVID-19 (right). (D) Relative expression patterns of representative genes in the trajectory analysis are plotted along the Pseudotime. The color indicates the relative gene expression calculated by Monocle 2. (E) Bar plots showing the average –log10(p*-*value) values in the enrichment analysis using the perturbed genes of four different cell lines in L1000 LINCS for up-regulated genes in cluster 3 (left) and cluster 1 (right). Error bars indicate standard deviation. (F) Comparison of combined enrichment scores between cluster 3 and cluster 1 for the gene sets from systemic lupus erythematosus (SLE) (n=16) and rheumatoid arthritis (RA) (n=5). ***p<0.001; ns, not significant. (G) GSEA of up-regulated genes in cluster 3 (left) and cluster 1 (right) to the ‘class 1’ gene module of monocyte-derived macrophages by Park *et al*. (2017). NES: normalized enrichment score, FDR: false discovery rate.

Next, we directly compared classical monocytes between mild and severe COVID-19. When we analyzed DEGs, severe COVID-19 was characterized by up-regulation of various ISGs, including *ISG15, IFITM1/2/3,* and *ISG20* (Fig. 5A). Both TNF/IL-1β-responsive genes and IFN-I-responsive genes were enriched in severe COVID-19-specific up-regulated genes (Fig. 5B). We measured plasma concentrations of TNF, IL-1β, IL-6, IFN-β, IFN-γ, and IL-18 in a larger cohort of COVID-19 patients. Among these cytokines, IL-6 and IL-18 were significantly increased in severe COVID-19 compared to mild COVID-19 whereas there was no difference in plasma concentrations of the other cytokines between the two groups (Fig. S5A). These results indicate that cytokine-responsive gene signatures cannot be simply explained by a few cytokines because of overlapped effects of cytokines.

To further investigate the characteristics of severe COVID-19, we performed a trajectory analysis with Monocle 2 (*23*) using two internally well-controlled specimens (one severe and one mild) in which both PBMC samples were collected from a single patient (the subject C7) with COVID-19. Trajectory analysis aligned classical monocytes along the disease severity with cluster 1 and cluster 3 corresponding to later and earlier Pseudotime, respectively (Fig. 5C). Representative genes in cluster 1 was enriched in the severe stage and highly associated with the both IFN-I and TNF/IL-1β-associated inflammatory response (Fig. 5D, Fig. S5B, and Table S11). GSEA confirmed that both the IFN-I response and TNF/IL-1β inflammatory response were prominent in cluster 1, but not in cluster 3 (Fig. 5E). Cluster 1 exhibited a significantly higher association with a gene set from systemic lupus erythematosus, which is a representative inflammatory disease with IFN-I features, than cluster 3 (Fig. 5F, left), but was not significantly associated with a gene set from rheumatoid arthritis (Fig. 5F, right).

We obtained additional evidence of the IFN-I-potentiated TNF inflammatory response in severe COVID-19 by analyzing a gene module that is not responsive to IFN-I, but associated with TNF-induced tolerance to TLR stimulation. Park *et al*. previously demonstrated that TNF tolerizes TLR-induced gene expression in monocytes, though TNF itself is an inflammatory cytokine (*24*). They also showed that IFN-I induces a hyper-inflammatory response by abolishing the tolerance effects of TNF, and defined a gene module responsible for the IFN-I-potentiated TNF-NF-κB inflammatory response as ‘class 1’ (*24*). This gene module was significantly enriched in cluster 1, but not in cluster 3 (Fig. 5G), which suggests that the IFN-I response may exacerbate hyper-inflammation by abolishing a negative feedback mechanism.

### Validation of hyperinflammatory features combined with IFN-I response in lung tissues from a lethal case of COVID-19

**Fig. 6:**
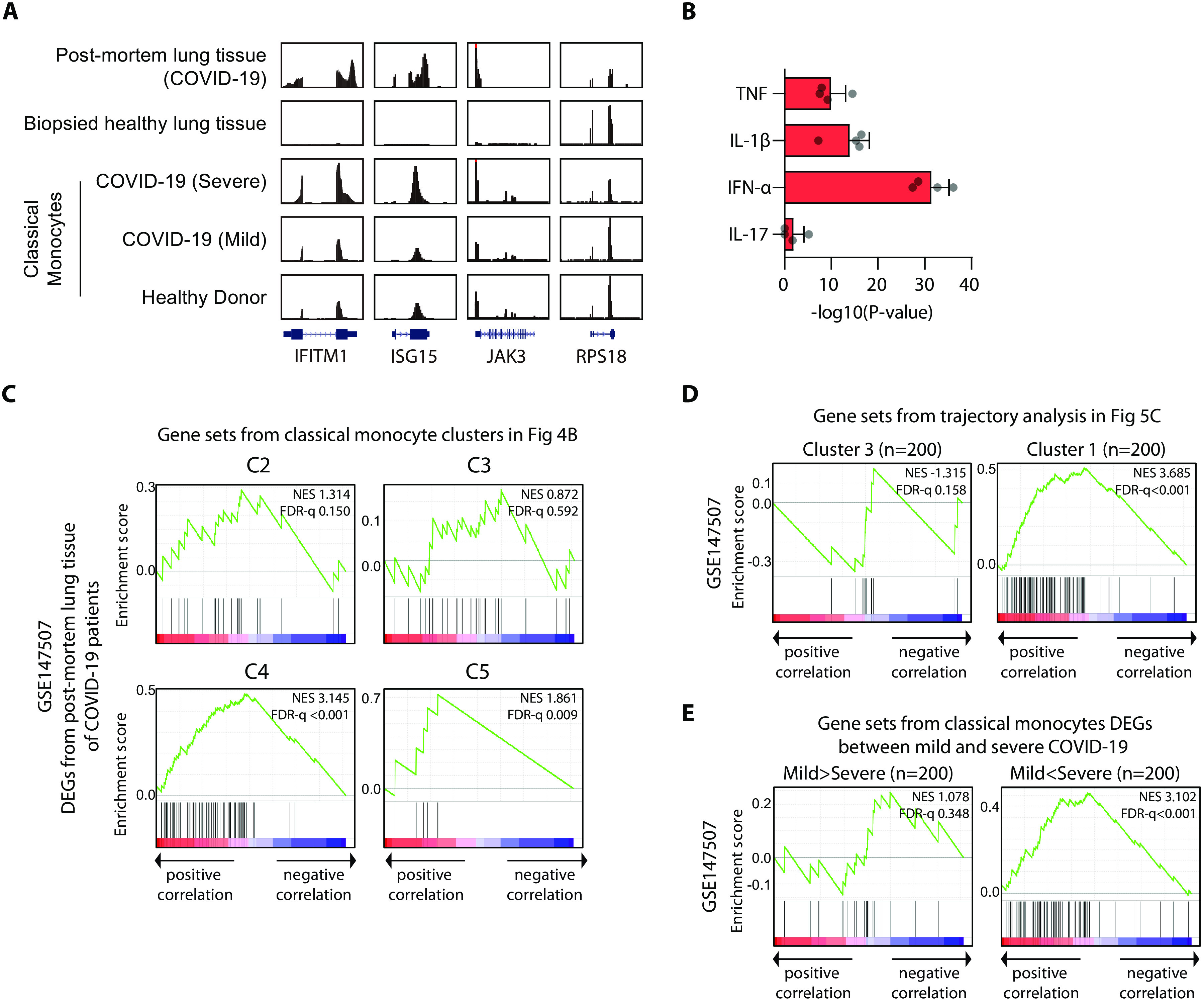
Validation of the combined IFN-I and inflammatory responses in the transcriptome of post-mortem lung tissues from lethal COVID-19. (A) UCSC Genome Browser snapshots of representative genes. (B) Bar plot showing the average –log10(p*-*value) values from the enrichment analysis using the perturbed genes of four different cell lines in L1000 LINCS for up-regulated genes (n= 386) in post-mortem lung tissues compared to biopsied healthy lung tissue. Error bars indicate standard deviation. (C) GSEA of significantly up- and down-regulated genes in post-mortem lung tissues for gene sets originated from up-regulated genes in C2 (n=96), C3 (n=143), C4 (n=218), and C5 (n=30) of Fig. 4B. (D and E) GSEA of significantly up- and down-regulated genes in post-mortem lung tissues for gene sets originated from the top 200 up-regulated genes in cluster 3 (left) and cluster 1 (right) from the trajectory analysis in Fig. 5C (D), and from gene sets originated from the top 200 up-regulated genes in classical monocytes of mild (left) and severe (right) COVID-19 (E).

Finally, we validated IFN-I response and inflammatory features using bulk RNA-seq data obtained using post-mortem lung tissues from patients with lethal COVID-19 (*25*). Although the analysis was limited to only two patients without individual cell-type resolution, in genome browser, up-regulation of *IFITM1, ISG15,* and *JAK3* and down-regulation of *RPS18* were observed commonly in post-mortem COVID-19 lung tissues and classical monocytes of severe COVID-19 (Fig. 6A). In the analysis with cytokine-responsive gene sets, both the IFN-I response and TNF/IL-1β-inflammatory response were prominent in the lung tissues (Fig. 6B). DEGs in the lung tissues were significantly associated with cluster 4, which is commonly up-regulated in both influenza and severe COVID-19, and cluster 5, which is specific to severe COVID-19 in Fig. 4B (Fig. 6C). These genes were also significantly associated with the cluster 1 identified in the trajectory analysis, but not with cluster 3 (Fig. 6D). When gene sets were defined by DEGs between mild and severe COVID-19, the DEGs in post-mortem lung tissues were significantly associated with genes up-regulated specifically in severe COVID-19 (Fig. 6E).

## DISCUSSION

Severe COVID-19 has been shown to be caused by a hyper-inflammatory response (*7*). Particularly, inflammatory cytokines secreted by classical monocytes and macrophages are considered to play a crucial role in severe progression of COVID-19 (*26*). In the current study, we confirmed the results from previous studies by showing that the TNF/IL-1β inflammatory response is dominant in COVID-19 although a small number of patients were enrolled. However, we also found that severe COVID-19 is accompanied by the IFN-I response in addition to the TNF/IL-1β response. These results indicate that the IFN-I response might contribute to the hyper-inflammatory response by potentiating TNF/IL-1β-driven inflammation in severe progression of COVID-19.

In the current study, we carried out scRNA-seq using PBMCs instead of specimens from the site of infection, e.g., lung tissues or bronchoalveolar lavage (BAL) fluids. However, hierarchical clustering based on relative changes to the healthy donor group showed that all types of cells among PBMCs were clustered together according to the disease groups as shown in Fig. 2A, indicating that there is disease-specific global impact across all types of cells among PBMCs. This finding suggests that peripheral blood immune cells are influenced by common inflammatory mediators regardless of cell type. However, we could not examine granulocytes in the current study because we used PBMCs, not whole blood samples for scRNA-seq.

In transcriptome studies for cytokine responses, we often analyze cytokine-responsive genes rather than cytokine genes themselves. However, we cannot exactly specify responsible cytokine(s) from the list of up-regulated genes because of overlapped effects of cytokines. For example, up-regulation of NF-κB-regulated genes can be driven by TNF, IL-1β or other cytokines, and up-regulation of IFN-responsive genes can be driven by IFN-I or other interferons. In the current study, we designated the IFN-I response because many up-regulated IFN-responsive genes were typical ISGs.

Recently, Wilk *et al*. also performed scRNA-seq using PBMCs from COVID-19 patients and healthy controls (*27*). Similar to our study, they found IFN-I-driven inflammatory signatures in monocytes from COVID-19 patients. However, they did not find substantial expression of pro-inflammatory cytokine genes such as *TNF*, *IL6*, *IL1B*, *CCL3*, *CCL4* and *CXCL2* in peripheral monocytes from COVID-19 patients whereas we detected the up-regulation of *TNF*, *IL1B*, *CCL3*, *CCL4* and *CXCL2* in the current study. Moreover, they found a developing neutrophil population in COVID-19 patients that was not detected in our study. These discrepant results might be due to different platforms for scRNA-seq. Wilk *et al*. used the Seq-Well platform whereas we used the 10X Genomics platform that is more generally used. We also note that recent scRNA-seq analyses of COVID-19 sometimes lead to unrelated or contradictory conclusions to each other despite the same platform (*28*, *29*). Although it often occurs in unsupervised analysis of highly multi-dimensional data, more caution will be required in designing scRNA-seq analysis of COVID-19, including definition of the severity and sampling time points.

Recently, Blanco-Melo *et al*. examined the transcriptional response to SARS-CoV-2 in in vitro infected cells, infected ferrets, and post-mortem lung samples from lethal COVID-19 patients and reported that IFN-I and -III responses are attenuated (*25*). However, we noted that IFN-I signaling pathway and innate immune response genes were relatively up-regulated in post-mortem lung samples from lethal COVID-19 patients compared to SARS-CoV-2-infected ferrets in their paper. Given that SARS-CoV-2 induces only mild disease without severe progression in ferrets (*30*), we interpret that IFN-I response is up-regulated in severe COVID-19 (e.g., post-mortem lung samples from lethal COVID-19 patients), but not in mild COVID-19 (e.g., SARS-CoV-2-infected ferrets). Indeed, severe COVID-19-specific signatures discovered in our current study were significantly enriched in the publically available data of post mortem lung tissues from the Blanco-Melo *et al*.’s study although the analysis was limited to only two patients without individual cell-type resolution (Fig. 6). In a recent study, Zhou *et al*. also found a robust IFN-I response in addition to pro-inflammatory response in BAL fluid of COVID-19 patients (*31*). Moreover, up-regulation of IFN-I-responsive genes has been demonstrated in SARS-CoV-2-infected intestinal organoids (*32*).

Although IFN-I has direct antiviral activity, their immunopathological role was also reported previously (*33*). In particular, the detrimental role of the IFN-I response was elegantly demonstrated in a murine model of SARS (*10*). In SARS-CoV-infected BALB/c mice, the IFN-I response induced the accumulation of pathogenic inflammatory monocytes-macrophages and vascular leakage, leading to death. It was proposed that a delayed, but considerable IFN-I response is critical for the development of acute respiratory distress syndrome and increased lethality during pathogenic coronavirus infection (*6*, *34*).

Currently, the management of patients with severe COVID-19 relies on intensive care and mechanical ventilation without a specific treatment because the pathogenic mechanisms of severe COVID-19 have not yet been clearly elucidated. In the current study, we demonstrated that severe COVID-19 is characterized by TNF/IL-1β-inflammatory features combined with the IFN-I response. In a murine model of SARS-CoV infection, timing of the IFN-I response is a critical factor determining outcomes of infection (*6*, *10*). Delayed IFN-I response contributes to pathological inflammation whereas early IFN-I response controls viral replication. Therefore, we propose that anti-inflammatory strategies targeting not only inflammatory cytokines, including TNF, IL-1β, and IL-6, but also pathological IFN-I response needs to be investigated for the treatment of patients with severe COVID-19.

## MATERIALS AND METHODS

### Patients

Patients diagnosed with COVID-19 were enrolled from Asan Medical Center, Severance Hospital, and Chungbuk National University Hospital. SARS-CoV-2 RNA was detected in patients’ nasopharyngeal swab and sputum specimens by multiplex real-time reverse-transcriptase PCR using the Allplex 2019-nCoV Assay kit (Seegene, Seoul, Republic of Korea). In this assay, N, RdRP, and E genes of SARS-CoV-2 were amplified, and Ct values were obtained for each gene. SARS-CoV-2-specific antibodies were examined using the SARS-CoV-2 Neutralization Antibody Detection kit (GenScript, Piscataway, NJ) and were positive in all COVID-19 patients in convalescent plasma samples or the last plasma sample in a lethal case. Hospitalized patients diagnosed with influenza A virus infection by a rapid antigen test of a nasopharyngeal swab were also enrolled from Asan Medical Center and Chungbuk National University Hospital from December 2015 to April 2016, prior to the emergence of COVID-19. Patients’ clinical features, laboratory findings, and chest radiographs were collected from their electronic medical records at each hospital. This study protocol was reviewed and approved by the institutional review boards of all participating institutions. Written informed consent was obtained from all patients.

### Single-cell RNA-seq

Peripheral blood mononuclear cells (PBMCs) were isolated from peripheral venous blood via standard Ficoll-Paque (GE Healthcare, Uppsala, Sweden) density gradient centrifugation, frozen in freezing media, and stored in liquid nitrogen until use. All samples showed a high viability of about 90% on average after thawing. Single-cell RNA-seq libraries were generated using the Chromium Single Cell 3′ Library & Gel Bead Kit v3 (10X genomics, Pleasanton, CA) following the manufacturer’s instructions. Briefly, thousands of cells were separated into nanoliter-scale droplets. In each droplet, cDNA was generated through reverse transcription. As a result, a cell barcoding sequence and Unique Molecular Identifier (UMI) were added to each cDNA molecule. Libraries were constructed and sequenced as a depth of approximately 50,000 reads per cell using the Nextseq 550 or Novaseq 6000 platform (Illumina, San Diego, CA).

### Single-cell RNA-seq data processing

The sequenced data were de-multiplexed using mkfastq (cellranger 10X genomics, v3.0.2) to generate fastq files. After de-multiplexing, the reads were aligned to the human reference genome (GRCh38; 10x cellranger reference GRCh38 v3.0.0), feature-barcode matrices generated using the cellranger count, and then aggregated by cellranger aggr using default parameters. The following analysis was performed using Seurat R package v3.1.5 (*17*). After generating the feature-barcode matrix, we discarded cells that expressed <200 genes and genes not expressed in any cells. To exclude low-quality cells from our data, we filtered out the cells that express mitochondrial genes in >15% of their total gene expression as described in previous studies (*29*, *35*, *36*). Doublets were also excluded, which were dominant in the cluster “Uncategorized 1”. Although there was a high variability in the number of UMIs detected per cell, majority of cells (90.5%) were enriched in a reasonable range of the UMIs (1,000 - 25,000), and 59% of cells with less than 1,000 UMIs were platelet or RBC excluded in downstream analysis. In each cell, the gene expression was normalized based on the total read count and log-transformed. To align the cells originating from different samples, 2000 highly variable genes from each sample were identified by the vst method in Seurat R package v3.1.5 (*17*). Using the canonical correlation analysis (CCA), we found anchors and aligned the samples based on the top 15 canonical correlation vectors. The aligned samples were scaled and principal component analysis (PCA) conducted. Finally, the cells were clustered by unsupervised clustering (0.5 resolution) and visualized by tSNE using the top 15 principal components.

### Cell type annotation through marker gene identification in each cluster

To identify marker genes, up-regulated genes in each cluster relative to the other clusters were selected based on the Wilcoxon rank sum test in Seurat’s implementation with >0.25 log fold change compared to the other clusters and a Bonferroni-adjusted p < 0.05 (Table S4). By manual inspection, among the 22 different clusters, 20 were assigned to 11 known immune cell types, RBCs which are characterized by *HBA1*, *HBA2*, and *HBB*, and platelets. The clusters characterized by similar marker genes were manually combined as one cell type. The two remaining clusters were assigned to ‘Uncategorized 1’ and ‘Uncategorized 2’ because they had no distinct features of known cell types. Based on the distribution of UMI counts, the cluster ‘Uncategorized 1’ was featured by relatively high UMIs per cell compared to other clusters and presence of higher expression of multiple cell type marker genes. The cluster ‘Uncategorized 2’ was featured by a B cell-like signatures and high expression of ribosomal protein genes, not recommended to be further analyzed according to the 10X platform guideline. In these aspects, RBCs, platelets, Uncategorized 1, and Uncategorized 2 were excluded in downstream analysis.

### Reproducibility of biological replicates

To check the reproducibility of biological replicates (individuals within a same group), we calculated the Spearman’s rank correlation coefficient for UMI counts that were merged according to each individual. The correlation coefficients of all individual pairs within the same group were visualized by a boxplot (COVID-19, n=45; FLU, n=10; HD, n=6).

### Hierarchical clustering of the transcriptomes at cell type resolution

In Fig. S1E, to investigate the similarity of the transcriptomes between cell types across diseases, we merged the UMI counts of each cell type according to healthy donor, influenza, mild COVID-19, and severe COVID-19. Next, the UMI counts for each gene were divided by the total UMI count in each cell type and multiplied by 100,000 as the normalized gene expression. Based on a median expression value >0.5, we calculated the relative changes in gene expression divided by the median value for each gene. Hierarchical clustering analysis was performed based on the PCC of the relative change in gene expression.

### Hierarchical clustering of variable gene expression among disease groups at cell type resolution

In Fig. 2A and Fig. S2A, to compare the highly variable gene expression among mild and severe COVID-19 and influenza relative to healthy donors, the normalized gene expression used in Fig. S1E was divided by the values in the healthy donor group. We selected the highly variable genes in terms of the top 25% standard deviation followed by log2-transformation (pseudo-count =1). In Fig. 2A, hierarchical clustering analysis was performed based on the PCCs of the selected highly variable genes. For Fig. S2A, to investigate the expression patterns of the selected highly variable genes (n=6,052), K-means clustering (k=50) was performed based on Euclidean distance. We manually ordered the clusters and visualized them as a heat map, revealing four distinct patterns: influenza-specific (n=1,046), COVID-19 specific (n=1,215), influenza/COVID-19 common (n=1,483), and cell type-specific (n=2,308).

### Analysis of dynamic changes in cell type composition compared to healthy donors

To investigate the dynamic changes in cell type composition, we calculated the proportion of cell types in each individual. As a control, we calculated the relative variation in each cell type composition between all pairs of healthy donors. Similarly, for each disease group, we calculated the relative variation in each cell type by dividing the fraction of the cell type in individual patient by that of individual healthy donor. After log2-transformation, we conducted statistical analysis using the relative variation in composition between the control and disease groups using a two-sided Kolmogorov–Smirnov test.

### Identification of DEGs using MAST

For any two transcriptome profiles, to identify DEGs, we utilized the model-based analysis of single cell transcriptomics (MAST) algorithm in Seurat’s implementation based on a Bonferroni-adjusted p<0.05 and a log2 fold change > 0.25.

### Gene Ontology analysis for biological pathways

In Fig. 2B, the DEGs in COVID-19 and influenza compared to healthy donors or COVID-19 compared to influenza were identified at cell type resolution. All DEGs were combined according to the disease groups for further analysis. The overlapping up or down DEGs between COVID-19 and influenza compared to healthy donors were defined as ‘Common up’ or ‘Common down’. The specific DEGs in COVID-19 or influenza were assigned as ‘COVID-19 up/down’ or ‘FLU up/down’, respectively. In addition, COVID-19-specific up- or down-regulated genes compared to influenza were assigned as ‘COVID-19>FLU’ or ‘FLU>COVID-19’, respectively. The Gene Ontology analysis was performed by DAVID. For each group of DEGs, the top 10 enriched GO biological pathways were selected, resulting in 49 unique GO biological pathways across all groups. The -log10(p-values) are shown as a heat map in Fig. 2B.

### WGCNA analysis to identify modular gene expression patterns

The weighted gene correlation network analysis (WGCNA) was conducted with the genes listed in the top 10 GO biological pathways of ‘COVID-19 up’, ‘FLU up’, and ‘Common up’ defined in Fig. 2B. The normalized gene expression values of the genes in COVID-19 were divided by the values in influenza and log2-transformed (pseudo-count =1). We used default parameters with the exception of soft threshold =10 and networkType = ‘signed’ when we constructed a topological overlap matrix. The modular gene expression patterns were defined using cutreeDynamic with a minClusterSize of 5. We visualized the modular gene expression pattern as a heat map in which the cell types were ordered according to hierarchical clustering with the default parameters of hcluster in R.

### Subclustering analysis

To find disease-specific subpopulations, each immune cell type was subjected to the subclustering analysis using Seurat. Briefly, the highly variable genes (n=1000) were selected based on vst and then scaled by ScaleData in Seurat with the vars.to.regress option to eliminate variation between individuals. The subpopulations were identified by FindClusters with default parameters, except resolution (non-EM-like CD8^+^ T cells, 0.3; classical monocytes, 0.2); the inputs were the top eight principal components (PCs) obtained from PCA of the scaled expression of the highly variable genes. The subpopulations were visualized by tSNE using the top eight PCs.

### Trajectory analysis

The trajectory analysis was performed with 2000 highly variable genes in classical monocytes across mild (C7-2) and severe (C7-1) COVID-19 as defined by the vst method in Seurat. The following analysis was performed using Monocle2. Briefly, the input was created from the UMI count matrix of the highly variable genes using the newCellDataSet function with default parameters, except expressionFamily = ‘negbinomial.size’. The size factors and dispersion of gene expression were estimated. The dimension of the normalized data was reduced based on DDRTree using reduceDimension with default parameters, except scaling = FALSE, which aligned the cells to the trajectory with three distinct clusters.

To determine genes that gradually changed along the trajectory, we identified the DEGs using MAST between clusters 1 and 3, which represent the severe stage and mild stage, respectively. The expression patterns of representative DEGs were visualized along the Pseudotime after correction with estimated size factors and dispersion for all genes.

### K-means clustering analysis of monocytes

In Fig. 4B, we performed K-means clustering of DEGs among all pairs of mild COVID-19, severe COVID-19, and influenza. The log2-transformed relative gene expression of DEGs compared to healthy donors was subjected to K-means clustering (k=10). Here, we used up-regulated DEGs in at least one disease group compared to the healthy donor group. We manually assigned five clusters based on gene expression patterns.

### Data analysis of the transcriptome profiles of post-mortem lung tissues

The transcriptome profiles of post-mortem lung tissues from two lethal cases of COVID-19 and biopsied heathy lung tissues from two donors were downloaded from a public database (GSE147507). The DEGs were identified using DESeq2 based on a Bonferroni-adjusted p < 0.05 and a log2 fold change > 1.

### Enrichment analysis using Enrichr and GSEA 4.0.3

Enrichr, the web-based software for gene set enrichment analysis (GSEA) was used for LINCS L1000 ligand perturbation analysis (*22*), virus perturbation analysis, and disease perturbation analysis from the GEO database. ‘Combined score’ was calculated as a parameter of enrichment as the log(p-value) multiplied by the z-score from the Fisher exact test. GSEA 4.0.3 software was used to conduct the GSEA when a ranked list of genes was available (Fig. 5G, Fig. 6C-E) (*37*). Results for IFN-γ-responsive genes were not presented because those were considerably overlapped with IFN-α-responsive genes, which are typical ISGs. The normalized enrichment score and FDR-q value were calculated to present the degree and significance of enrichment.

### Flow cytometry analysis

Cryopreserved PBMCs were thawed, and dead cells were stained using the Live/Dead Fixable Cell Stain kit (Invitrogen, Carlsbad, CA). Cells were stained with fluorochrome-conjugated antibodies, including anti-CD3 (BV605; BD Biosciences), anti-CD4 (BV510; BD Biosciences), anti-CD8 (BV421; BD Biosciences), anti-CD14 (PE-Cy7; BD Biosciences), anti-CD19 (Alexa Fluor 700; BD Biosciences), and anti-CD56 (VioBright FITC; Miltenyi Biotec). For staining with anti-granzyme B (BD Biosciences), cells were permeabilized using a Foxp3 staining buffer kit (eBioscience).

For intracellular cytokine staining of IFN-γ, PBMCs were stimulated with phorbol 12-myristate 13-acetate (PMA, 50 ng/ml) (Sigma Aldrich) and ionomycin (1 μg/ml) (Sigma Aldrich). Brefeldin A (GolgiPlug, BD Biosciences) and monesin (GolgiStop, BD Biosciences) were added 1 hour later. After another 5 hours of incubation, cells were harvested for staining with the Live/Dead Fixable Cell Stain kit, anti-CD3, anti-CD4, and anti-CD8. Following cell permeabilization, cells were further stained with anti-IFN-γ (Alexa Fluor 488; eBioscience).

Flow cytometry was performed on an LSR II instrument using FACSDiva software (BD Biosciences) and the data analyzed using FlowJo software (Treestar, San Carlos, CA).

### ELISA and cytometric bead arrays

Cytokines were measured in plasma samples, including IFN-β, IL-18 (ELISA, R&D Systems, Minneapolis, MN), IL-1β (Cytometric bead array flex kit, BD Biosciences, San Jose, CA), TNF, IL-6, and IFN-γ (LEGENDplex bead-based immunoassay kit, BioLegend, San Diego, CA).

### Statistical analysis

We performed the KS test to compare the distributions of two groups without assuming that the distributions follow normality. Welch’s *t* test was conducted to compare the two distributions after confirming the normality of the distributions using the Shapiro-Wilk normality test. A Wilcoxon signed rank test was conducted to compare the differences between two groups with paired subjects. The Mann-Whitney test was performed to compare the means of two groups. Statistical analyses were performed using Prism software version 5.0 (GraphPad, La Jolla, CA). p<0.05 was considered significant.

## Supplementary Materials

immunology.sciencemag.org/cgi/content/full/5/49/eabd1554/DC1 Fig. S1. Clinical characteristics and assessment of the quality of scRNA-seq results. Fig. S2. Transcriptome features of highly variable genes. Fig. S3. Characterization of disease-specific CD8^+^ T-cell subpopulations. Fig. S4. Subpopulation analysis of classical monocytes. Fig. S5. STRING analysis of up-regulated genes in cluster 1 obtained from the trajectory analysis of classical monocytes. Table S1. Experimental batches of scRNA-seq. Table S2. Clinical characteristics of severe influenza patients. Table S3. Clinical characteristics of COVID-19 patients. Table S4. The scRNA-seq results. Table S5. A list of marker genes for each cluster. Table S6. A list of DEGs and associated biological pathways in Fig. 2B. Table S7. Cell types in which the GBP1, CREM, and CCL3 were upregulated in Fig. 2C. Table S8. A list of genes in each module obtained from WGCNA in Fig. 2D. Table S9. A list of up-regulated genes in non-EM-like CD8^+^ T-cell subpopulations. Table S10. A list of genes included in each cluster defined by K-mean clustering of classical monocytes. Table S11. A list of genes up-regulated in early and late Pseudotime.

## References

[1] Wu F., Zhao S., Yu B., Chen Y. M., Wang W., Song Z. G., Hu Y., Tao Z. W., Tian J. H., Pei Y. Y., Yuan M. L., Zhang Y. L., Dai F. H., Liu Y., Wang Q. M., Zheng J. J., Xu L., Holmes E. C., Zhang Y. Z., A new coronavirus associated with human respiratory disease in China. Nature 579, 265–269 (2020). 10.1038/s41586-020-2008-3 32015508 PMC7094943

[2] Zhu N., Zhang D., Wang W., Li X., Yang B., Song J., Zhao X., Huang B., Shi W., Lu R., Niu P., Zhan F., Ma X., Wang D., Xu W., Wu G., Gao G. F., Tan W.; China Novel Coronavirus Investigating and Research Team, A Novel Coronavirus from Patients with Pneumonia in China, 2019. N. Engl. J. Med. 382, 727–733 (2020). 10.1056/NEJMoa2001017 31978945 PMC7092803

[3] World Health Organization, Coronavirus disease 2019 (COVID-19) Situation Report –134, June 2, 2020; https://www.who.int/docs/default-source/coronaviruse/situation-reports/20200602-covid-19-sitrep-134.pdf?sfvrsn=cc95e5d5_2 (2020).

[4] Guan W. J., Ni Z. Y., Hu Y., Liang W. H., Ou C. Q., He J. X., Liu L., Shan H., Lei C. L., Hui D. S. C., Du B., Li L. J., Zeng G., Yuen K. Y., Chen R. C., Tang C. L., Wang T., Chen P. Y., Xiang J., Li S. Y., Wang J. L., Liang Z. J., Peng Y. X., Wei L., Liu Y., Hu Y. H., Peng P., Wang J. M., Liu J. Y., Chen Z., Li G., Zheng Z. J., Qiu S. Q., Luo J., Ye C. J., Zhu S. Y., Zhong N. S.; China Medical Treatment Expert Group for Covid-19, Clinical Characteristics of Coronavirus Disease 2019 in China. N. Engl. J. Med. 382, 1708–1720 (2020). 10.1056/NEJMoa2002032 32109013 PMC7092819

[5] Wu J. T., Leung K., Bushman M., Kishore N., Niehus R., de Salazar P. M., Cowling B. J., Lipsitch M., Leung G. M., Estimating clinical severity of COVID-19 from the transmission dynamics in Wuhan, China. Nat. Med. 26, 506–510 (2020). 10.1038/s41591-020-0822-7 32284616 PMC7094929

[6] Channappanavar R., Perlman S., Pathogenic human coronavirus infections: Causes and consequences of cytokine storm and immunopathology. Semin. Immunopathol. 39, 529–539 (2017). 10.1007/s00281-017-0629-x 28466096 PMC7079893

[7] Pedersen S. F., Ho Y. C., SARS-CoV-2: A storm is raging. J. Clin. Invest. 130, 2202–2205 (2020). 10.1172/JCI137647 32217834 PMC7190904

[8] Chen G., Wu D., Guo W., Cao Y., Huang D., Wang H., Wang T., Zhang X., Chen H., Yu H., Zhang X., Zhang M., Wu S., Song J., Chen T., Han M., Li S., Luo X., Zhao J., Ning Q., Clinical and immunological features of severe and moderate coronavirus disease 2019. J. Clin. Invest. 130, 2620–2629 (2020). 10.1172/JCI137244 32217835 PMC7190990

[9] Ong E. Z., Chan Y. F. Z., Leong W. Y., Lee N. M. Y., Kalimuddin S., Haja Mohideen S. M., Chan K. S., Tan A. T., Bertoletti A., Ooi E. E., Low J. G. H., A Dynamic Immune Response Shapes COVID-19 Progression. Cell Host Microbe 27, 879–882.e2 (2020). 10.1016/j.chom.2020.03.021 32359396 PMC7192089

[10] Channappanavar R., Fehr A. R., Vijay R., Mack M., Zhao J., Meyerholz D. K., Perlman S., Dysregulated Type I Interferon and Inflammatory Monocyte-Macrophage Responses Cause Lethal Pneumonia in SARS-CoV-Infected Mice. Cell Host Microbe 19, 181–193 (2016). 10.1016/j.chom.2016.01.007 26867177 PMC4752723

[11] Qin C., Zhou L., Hu Z., Zhang S., Yang S., Tao Y., Xie C., Ma K., Shang K., Wang W., Tian D. S., Dysregulation of immune response in patients with COVID-19 in Wuhan, China. Clin. Infect. Dis. ciaa248 (2020). 10.1093/cid/ciaa248 32161940 PMC7108125

[12] Zheng H. Y., Zhang M., Yang C. X., Zhang N., Wang X. C., Yang X. P., Dong X. Q., Zheng Y. T., Elevated exhaustion levels and reduced functional diversity of T cells in peripheral blood may predict severe progression in COVID-19 patients. Cell. Mol. Immunol. 17, 541–543 (2020). 10.1038/s41423-020-0401-3 32203186 PMC7091621

[13] Zheng M., Gao Y., Wang G., Song G., Liu S., Sun D., Xu Y., Tian Z., Functional exhaustion of antiviral lymphocytes in COVID-19 patients. Cell. Mol. Immunol. 17, 533–535 (2020). 10.1038/s41423-020-0402-2 32203188 PMC7091858

[14] Xu Z., Shi L., Wang Y., Zhang J., Huang L., Zhang C., Liu S., Zhao P., Liu H., Zhu L., Tai Y., Bai C., Gao T., Song J., Xia P., Dong J., Zhao J., Wang F. S., Pathological findings of COVID-19 associated with acute respiratory distress syndrome. Lancet Respir. Med. 8, 420–422 (2020). 10.1016/S2213-2600(20)30076-X 32085846 PMC7164771

[15] Giamarellos-Bourboulis E. J., Netea M. G., Rovina N., Akinosoglou K., Antoniadou A., Antonakos N., Damoraki G., Gkavogianni T., Adami M. E., Katsaounou P., Ntaganou M., Kyriakopoulou M., Dimopoulos G., Koutsodimitropoulos I., Velissaris D., Koufargyris P., Karageorgos A., Katrini K., Lekakis V., Lupse M., Kotsaki A., Renieris G., Theodoulou D., Panou V., Koukaki E., Koulouris N., Gogos C., Koutsoukou A., Complex Immune Dysregulation in COVID-19 Patients with Severe Respiratory Failure. Cell Host Microbe 27, 992–1000.e3 (2020). 10.1016/j.chom.2020.04.009 32320677 PMC7172841

[16] Smith G. B., Prytherch D. R., Meredith P., Schmidt P. E., Featherstone P. I., The ability of the National Early Warning Score (NEWS) to discriminate patients at risk of early cardiac arrest, unanticipated intensive care unit admission, and death. Resuscitation 84, 465–470 (2013). 10.1016/j.resuscitation.2012.12.016 23295778

[17] Butler A., Hoffman P., Smibert P., Papalexi E., Satija R., Integrating single-cell transcriptomic data across different conditions, technologies, and species. Nat. Biotechnol. 36, 411–420 (2018). 10.1038/nbt.4096 29608179 PMC6700744

[18] Finak G., McDavid A., Yajima M., Deng J., Gersuk V., Shalek A. K., Slichter C. K., Miller H. W., McElrath M. J., Prlic M., Linsley P. S., Gottardo R., MAST: A flexible statistical framework for assessing transcriptional changes and characterizing heterogeneity in single-cell RNA sequencing data. Genome Biol. 16, 278 (2015). 10.1186/s13059-015-0844-5 26653891 PMC4676162

[19] Langfelder P., Horvath S., WGCNA: An R package for weighted correlation network analysis. BMC Bioinformatics 9, 559 (2008). 10.1186/1471-2105-9-559 19114008 PMC2631488

[20] Mitchell H. D., Eisfeld A. J., Sims A. C., McDermott J. E., Matzke M. M., Webb-Robertson B. J., Tilton S. C., Tchitchek N., Josset L., Li C., Ellis A. L., Chang J. H., Heegel R. A., Luna M. L., Schepmoes A. A., Shukla A. K., Metz T. O., Neumann G., Benecke A. G., Smith R. D., Baric R. S., Kawaoka Y., Katze M. G., Waters K. M., A network integration approach to predict conserved regulators related to pathogenicity of influenza and SARS-CoV respiratory viruses. PLOS ONE 8, e69374 (2013). 10.1371/journal.pone.0069374 23935999 PMC3723910

[21] Szklarczyk D., Gable A. L., Lyon D., Junge A., Wyder S., Huerta-Cepas J., Simonovic M., Doncheva N. T., Morris J. H., Bork P., Jensen L. J., Mering C. V., STRING v11: Protein-protein association networks with increased coverage, supporting functional discovery in genome-wide experimental datasets. Nucleic Acids Res. 47 (D1), D607–D613 (2019). 10.1093/nar/gky1131 30476243 PMC6323986

[22] Duan Q., Flynn C., Niepel M., Hafner M., Muhlich J. L., Fernandez N. F., Rouillard A. D., Tan C. M., Chen E. Y., Golub T. R., Sorger P. K., Subramanian A., Ma’ayan A., LINCS Canvas Browser: Interactive web app to query, browse and interrogate LINCS L1000 gene expression signatures. Nucleic Acids Res. 42 (W1), W449–W460 (2014). 10.1093/nar/gku476 24906883 PMC4086130

[23] Qiu X., Hill A., Packer J., Lin D., Ma Y. A., Trapnell C., Single-cell mRNA quantification and differential analysis with Census. Nat. Methods 14, 309–315 (2017). 10.1038/nmeth.4150 28114287 PMC5330805

[24] Park S. H., Kang K., Giannopoulou E., Qiao Y., Kang K., Kim G., Park-Min K. H., Ivashkiv L. B., Type I interferons and the cytokine TNF cooperatively reprogram the macrophage epigenome to promote inflammatory activation. Nat. Immunol. 18, 1104–1116 (2017). 10.1038/ni.3818 28825701 PMC5605457

[25] Blanco-Melo D., Nilsson-Payant B. E., Liu W. C., Uhl S., Hoagland D., Møller R., Jordan T. X., Oishi K., Panis M., Sachs D., Wang T. T., Schwartz R. E., Lim J. K., Albrecht R. A., tenOever B. R., I, Imbalanced Host Response to SARS-CoV-2 Drives Development of COVID-19. Cell 181, 1036–1045.e9 (2020). 10.1016/j.cell.2020.04.026 32416070 PMC7227586

[26] Merad M., Martin J. C., Pathological inflammation in patients with COVID-19: A key role for monocytes and macrophages. Nat. Rev. Immunol. 20, 355–362 (2020). 10.1038/s41577-020-0331-4 32376901 PMC7201395

[27] Wilk A. J., Rustagi A., Zhao N. Q., Roque J., Martínez-Colón G. J., McKechnie J. L., Ivison G. T., Ranganath T., Vergara R., Hollis T., Simpson L. J., Grant P., Subramanian A., Rogers A. J., Blish C. A., A single-cell atlas of the peripheral immune response in patients with severe COVID-19. Nat. Med. (2020). 10.1038/s41591-020-0944-y 32514174 PMC7382903

[28] Liao M., Liu Y., Yuan J., Wen Y., Xu G., Zhao J., Cheng L., Li J., Wang X., Wang F., Liu L., Amit I., Zhang S., Zhang Z., Single-cell landscape of bronchoalveolar immune cells in patients with COVID-19. Nat. Med. 26, 842–844 (2020). 10.1038/s41591-020-0901-9 32398875

[29] Chua R. L., Lukassen S., Trump S., Hennig B. P., Wendisch D., Pott F., Debnath O., Thürmann L., Kurth F., Völker M. T., Kazmierski J., Timmermann B., Twardziok S., Schneider S., Machleidt F., Müller-Redetzky H., Maier M., Krannich A., Schmidt S., Balzer F., Liebig J., Loske J., Suttorp N., Eils J., Ishaque N., Liebert U. G., von Kalle C., Hocke A., Witzenrath M., Goffinet C., Drosten C., Laudi S., Lehmann I., Conrad C., Sander L.-E., Eils R., COVID-19 severity correlates with airway epithelium-immune cell interactions identified by single-cell analysis. Nat. Biotechnol. (2020). 10.1038/s41587-020-0602-4 32591762

[30] Kim Y. I., Kim S. G., Kim S. M., Kim E. H., Park S. J., Yu K. M., Chang J. H., Kim E. J., Lee S., Casel M. A. B., Um J., Song M. S., Jeong H. W., Lai V. D., Kim Y., Chin B. S., Park J. S., Chung K. H., Foo S. S., Poo H., Mo I. P., Lee O. J., Webby R. J., Jung J. U., Choi Y. K., Infection and Rapid Transmission of SARS-CoV-2 in Ferrets. Cell Host Microbe 27, 704–709.e2 (2020). 10.1016/j.chom.2020.03.023 32259477 PMC7144857

[31] Zhou Z., Ren L., Zhang L., Zhong J., Xiao Y., Jia Z., Guo L., Yang J., Wang C., Jiang S., Yang D., Zhang G., Li H., Chen F., Xu Y., Chen M., Gao Z., Yang J., Dong J., Liu B., Zhang X., Wang W., He K., Jin Q., Li M., Wang J., Heightened Innate Immune Responses in the Respiratory Tract of COVID-19 Patients. Cell Host Microbe 27, 883–890.e2 (2020). 10.1016/j.chom.2020.04.017 32407669 PMC7196896

[32] Lamers M. M., Beumer J., van der Vaart J., Knoops K., Puschhof J., Breugem T. I., Ravelli R. B. G., Paul van Schayck J., Mykytyn A. Z., Duimel H. Q., van Donselaar E., Riesebosch S., Kuijpers H. J. H., Schipper D., van de Wetering W. J., de Graaf M., Koopmans M., Cuppen E., Peters P. J., Haagmans B. L., Clevers H., SARS-CoV-2 productively infects human gut enterocytes. Science 369, 50–54 (2020). 10.1126/science.abc1669 32358202 PMC7199907

[33] Davidson S., Maini M. K., Wack A., Disease-promoting effects of type I interferons in viral, bacterial, and coinfections. J. Interferon Cytokine Res. 35, 252–264 (2015). 10.1089/jir.2014.0227 25714109 PMC4389918

[34] Kindler E., Thiel V., SARS-CoV and IFN: Too Little, Too Late. Cell Host Microbe 19, 139–141 (2016). 10.1016/j.chom.2016.01.012 26867172 PMC7104995

[35] Maier B., Leader A. M., Chen S. T., Tung N., Chang C., LeBerichel J., Chudnovskiy A., Maskey S., Walker L., Finnigan J. P., Kirkling M. E., Reizis B., Ghosh S., D’Amore N. R., Bhardwaj N., Rothlin C. V., Wolf A., Flores R., Marron T., Rahman A. H., Kenigsberg E., Brown B. D., Merad M., A conserved dendritic-cell regulatory program limits antitumour immunity. Nature 580, 257–262 (2020). 10.1038/s41586-020-2134-y 32269339 PMC7787191

[36] Wei K., Korsunsky I., Marshall J. L., Gao A., Watts G. F. M., Major T., Croft A. P., Watts J., Blazar P. E., Lange J. K., Thornhill T. S., Filer A., Raza K., Donlin L. T., Siebel C. W., Buckley C. D., Raychaudhuri S., Brenner M. B.; Accelerating Medicines Partnership Rheumatoid Arthritis & Systemic Lupus Erythematosus (AMP RA/SLE) Consortium, Notch signalling drives synovial fibroblast identity and arthritis pathology. Nature 582, 259–264 (2020). 10.1038/s41586-020-2222-z 32499639 PMC7841716

[37] Mootha V. K., Lindgren C. M., Eriksson K. F., Subramanian A., Sihag S., Lehar J., Puigserver P., Carlsson E., Ridderstråle M., Laurila E., Houstis N., Daly M. J., Patterson N., Mesirov J. P., Golub T. R., Tamayo P., Spiegelman B., Lander E. S., Hirschhorn J. N., Altshuler D., Groop L. C., PGC-1alpha-responsive genes involved in oxidative phosphorylation are coordinately downregulated in human diabetes. Nat. Genet. 34, 267–273 (2003). 10.1038/ng1180 12808457

